# Deciphering the Effects of Phosphate Fertiliser on Rhizospheric Bacterial Community Structure and Potato Common Scab

**DOI:** 10.3390/microorganisms12112322

**Published:** 2024-11-15

**Authors:** Shanshan Chen, Jingjing Cao, Pan Zhao, Zhiqin Wang, Xiu Wang, Genhong Liu, Naiqin Zhong

**Affiliations:** 1 School of Agriculture, Ningxia University, Yinchuan 750021, China; css6867@163.com; 2 State Key Laboratory of Plant Genomics, Institute of Microbiology, Chinese Academy of Sciences, Beijing 100101, China; m18409483820@163.com (J.C.); zhaop@im.ac.cn (P.Z.); wangzq1210@163.com (Z.W.); 3 Engineering Laboratory for Advanced Microbial Technology of Agriculture, Chinese Academy of Sciences, Beijing 100101, China; 4 Key Laboratory of Potato Industry Integration and Development Enterprises in Inner Mongolia Autonomous Region, Hulunbuir 021000, China; 5 Hulunbuir Agricultural and Animal Husbandry Technology Promotion Center, Hulunbuir 021000, China; wangxiu1971@163.com

**Keywords:** different habitats, phosphate application rate, bacterial community diversity, antagonistic bacteria, *S. scabies*

## Abstract

The prolonged practice of continuous potato cropping, coupled with inadequate field management, disrupts the soil bacterial community equilibrium. Such disturbances compromise the resilience of the soil ecosystem, predisposing it to an increased incidence of potato diseases. However, the effects of the phosphorus fertiliser application rate on the rhizosphere soil bacterial community composition of potatoes and the occurrence of potato common scab (CS) have not been adequately studied. Here, diseased field soils from Dingxi and Huidong Counties were collected for potting tests, and field tests were conducted in Huidong County for validation. An examination of the relationship between the bacterial community composition in the potato rhizosphere soil and potato CS under different phosphate fertiliser treatments was conducted using 16S rRNA high-throughput sequencing. The results show that a lower phosphorus fertiliser application rate was more conducive to maintaining soil bacterial community diversity under different phosphorus fertiliser treatments in different habitats. In addition, the relative abundance of the *txtA* gene increased significantly (*p* < 0.05) with the increase in the phosphate fertiliser application rate. Field trials conducted in Huidong revealed that treatments F1, F2, and F3 had respective CS incidence rates of 28.33%, 46.67%, and 59.44%, while their corresponding disease severity indices were 7.67, 17.33, and 29.44. Further analysis revealed that the relative abundance of antagonistic genera of pathogenic *S. scabies* decreased significantly (*p* < 0.05) with increases in the phosphorus fertiliser application rate. In summary, the correlation between potato CS and changes in the bacterial community of rhizosphere soil was used to determine the optimal phosphorus application rate during potato production, which can provide a scientific basis for the management of phosphorus fertiliser in potato farmland.

## 1. Introduction

Potatoes are the fourth largest staple crop after wheat, corn, and rice, with their total production exceeding 3.68 billion tons [1]. They play an important role in maintaining global food security and supporting population growth [2]. Potatoes are sensitive to nutrients, and fertiliser application is necessary to ensure high and stable yields [3]; however, fertiliser is often applied in excess and unevenly by growers in pursuit of better efficiency [4], resulting in lower fertiliser utilisation rates [5]. Meanwhile, the uneven distribution of nutrients in the soil leads to the gradual deterioration of the soil’s microecology, resulting in an increasing trend of soil-borne diseases year by year [6]. In recent years, potato CS caused by pathogenic *Streptomyces* spp. has spread globally. Over 20 species of pathogens have been reported [7], with diverse transmission routes and differential distribution across multiple potato ecological production areas [8]. This issue has become a pivotal constraint on the sustainable development of the potato industry, particularly impacting the seed potato sector due to the significant challenges it presents for prevention and control measures. Thaxtomin A is the main virulence factor of pathogenic *Streptomyces* spp., with its biosynthetic genes including *txtA*, *txtB*, *txtC*, *txtD*, *txtE*, *txtH*, and txtR, which play pivotal roles in the biosynthetic pathways and pathogenicity of these organisms [9]. Among these, *txtA* is frequently utilised as a pivotal gene for quantifying the pathogenicity of *Streptomyces* in soil [10], enabling the assessment of the relative abundance of pathogenic *Streptomyces* species; consequently, this approach provides a suite of methods for predicting and controlling potato CS disease.

Phosphorus (P), integral to the structure of biological macromolecules, including DNA, RNA, ATP, and phospholipids, plays a pivotal role in facilitating rapid leaf expansion, root cell division, and starch synthesis as well as providing lubrication within cells [11]. As an indispensable nutrient for crop growth and development, enhancing the application rate of phosphorus fertilisers stands out as a key strategy for boosting crop productivity. Potatoes are shallow-rooted crops [12] and the recovery rate of phosphorus is only 10–15% [13]. This results in most of the phosphorus in the fertiliser combining with Ca^2+^, Fe^2+^, Fe^3+^, and Al^3+^ in the soil to form insoluble phosphates that are not absorbed and utilised by the plant, increasing their content [14] and causing serious impacts on the environment. Applying large amounts of phosphate fertilisers to farmland may deplete beneficial microorganisms and increase the relative abundance of harmful microorganisms [15]. Therefore, scientifically determining the application rate of phosphorus fertilisers is very important. Many scholars have focused on the amount of phosphorus fertiliser that matches the highest yield and best economic performance of potatoes [16] but the effects of phosphorus fertiliser deposition on the diversity of soil bacterial communities in the rhizosphere of potatoes, as well as on soil-borne diseases, have not yet been adequately investigated. For example, it was found that the amount of phosphorus fertiliser was negatively correlated with the incidence and extent of potato CS [17]; however, recently, it was found that the severity of CS was positively correlated with the amount of phosphate fertiliser [18]. Therefore, determining the appropriate amount of phosphate fertiliser for potato field management is of great significance for the prevention and control of potato CS and the development of soil microecology.

Soil microorganisms play a key role in the phosphorus cycle and are environmentally friendly and inexpensive tools for increasing soil-effective phosphorus. As an important component of soil microorganisms, changes in the community structure of plant rhizosphere microorganisms have a direct impact on the soil nutrient environment and plant health [19]. In particular, bacteria are very sensitive to mineral fertiliser inputs [20] and can more directly reflect changes in the soil nutrient status. Therefore, observing changes in the relative abundance of bacteria can be effective in assessing the health of soil ecosystems. The high-throughput sequencing of microbial 16S rRNA gene amplicons is an effective approach for studying changes in the microbial community structure in different environments. Sequencing can not only reveal patterns of microbial community diversity but also reflect the microbial community composition and structure and can predict microbial function. Microbial communities with different functions participate in various processes in the C, N, and S cycles, which can help maintain ecosystem stability to some extent [21]. Furthermore, the response of soil microbial communities to phosphorus fertiliser management in dry-crop farmland was investigated using 16S rRNA amplicon sequencing, and it was found that increased phosphorus fertiliser application rates reduced the diversity of soil bacteria and fungi, species evenness, and the abundance of key genera in wheat fields [22]. Additionally, long-term phosphorus fertiliser application rates affect soil bacterial and microbial diversity in alfalfa fields and can lead to reduced microbial activity [23].

To date, there have been no reports on how phosphate fertilisers alter the bacterial community structure in potato rhizosphere soil and their subsequent impact on the severity of potato CS. In this study, it is hypothesised that the application of phosphate fertiliser significantly alters the bacterial community structure in the rhizosphere of potatoes, potentially exacerbating the incidence and severity of potato scab. To test this hypothesis, we employed high-throughput sequencing of 16S rRNA gene amplicons to investigate the response of the soil bacterial community structure to varying rates of phosphate fertiliser application. The aim of this study was to analyse the impact of the phosphate application rate on the abundance of *Streptomyces scabies* and its antagonistic bacteria in potato rhizosphere soils.

## 2. Materials and Methods

### 2.1. Experimental Methods

#### 2.1.1. Pot Experiment

A pot experiment was conducted using soil from potato CS disease fields with long-term continuous cropping as the cultivation substrate. Samples were collected from Huidong County, Guangdong Province, China (22°98′ N, 114°72′ E), and Dingxi City, Gansu Province (35°58′ N, 104°62′ E). The experiment consisted of three different gradients of phosphorus fertiliser treatments on the field-collected soils, namely, low P (no additional P_2_O_5_), medium P (120 kg/ha of P_2_O_5_ applied), and high P (240 kg/ha of P_2_O_5_ applied). These treatments were labelled as soil samples coded DXG1 (low P), DXG2 (medium P), and DXG3 (high P) in Dingxi pots and HDG1 (low P), HDG2 (medium P), and HDG3 (high P) in Huidong pots, respectively. All fertilisers were applied at once before sowing; the specific application rates are shown in Table 1. The susceptible potato variety ‘shepody’ was used as the test plant, and 3 detoxified test tube seedlings were planted in each pot with a diameter of 17 cm and a height of 10 cm. The pot experiments were conducted under the conditions of a temperature of 25 °C, a light intensity of 50,000 Lux, and a 16 h/8 h light cycle, with each treatment repeated three times. The relative chlorophyll content (SPAD-502 chlorophyll meter, with the relative content expressed as SPAD), plant height, and stem diameter were determined at the potato tuberisation stage. After 100 d of cultivation, potato rhizosphere soil samples were collected for amplification and sequencing of the 16S rRNA gene segment.

#### 2.1.2. Field Experiment

A field experiment was conducted in Huidong County, Guangdong Province. Similar to the pot experiment, three different gradients of phosphorus fertiliser treatments were used as follows: HDF1 (low P), HDF2 (medium P), and HDF3 (high P). The fertiliser application rates are shown in Table 1. The nutrient management method was the same as that used in the pot experiment, with each treatment repeated three times. Ridge planting was carried out with double rows, a community area of 18 m^2^, and a planting density of 75,000 plants/ha. Using the multi-point sampling method, soil samples were collected from the surface of each treated tuber [24] and 16Sr RNA gene fragment amplicon sequencing and analysis were performed. After 100 days of growth, 60 tubers were randomly selected from each treatment for disease severity assessment using the following calculation method:

Disease rate (%) = Number of diseased tubers/total number of surveyed tubers × 100

Disease index = [∑ (n_1 × 1 + n_2 × 2 + n_3 × 3 + n_4 × 4 + n_5 × 5)/ (N × 5)] × 100

where n = the number of tubers corresponding to the numerical grade; N = the total number of potato tubers assessed; 5 = a high score on the severity scale.

The percentage of tuber area covered was rated as follows: (0) no symptoms of scab; (1) 0–12.5%; (2) 12.6–25%; (3) 26–50%; (4) 51–75%; (5) 76–100%.

### 2.2. Illumina MiSeq Sequencing

#### 2.2.1. Soil Sample DNA Extraction and High-Throughput Sequencing

The total DNA was extracted from the rhizosphere soil samples using the Magen Soil DNA Kit from Guangzhou, China. The extracted genomic DNA was used as the template to amplify the V3–V4 region of the 16S rRNA genes with the 515 forward (5′-GTGCCAGCMGCCGCGGTAA-3′) and 806 reverse (5′-GGACTACHVGGGTWTCTAAT-3′) primers [25]. The PCR product was separated via agarose gel electrophoresis and purified. An Illumina PE250 high-throughput sequencing platform was used to generate the raw sequencing data. The library construction and sequencing were conducted by Novogene Co., Ltd. (Novogene Co., Ltd., Beijing, China). The sequencing library was built using a TruSeq^®^ DNA PCR-Free Sample Preparation Kit (Illumina, San Diego, CA, USA). The DNA concentration was measured using a Qubit^®^ dsDNA Assay Kit on a Qubit^®^ 2.0 Fluorometer (Life Technologies, Carlsbad, CA, USA). Once the libraries passed quality control, they were sequenced on the NovaSeq6000 platform.

#### 2.2.2. Species Annotation and Diversity Analysis

The raw sequencing data from this study were subjected to both OTU and ASV analyses to comprehensively assess the bacterial community diversity. Overlapping reads were merged using the program FLASH with the default parameters. QIIME II (version 1.9.1) [26] and UCHIME [27] were used and the effective tags were obtained. The raw sequences were filtered, denoised, and merged, and the chimeric sequences were removed using the DATA2 plugin, followed by the annotation of each amplicon sequence variant (ASV). We employed UPARSE (version 7.0.1001) [28] to cluster sequences into operational taxonomic units (OTUs) based on an open-reference OTU picking protocol with a 97% nucleotide similarity threshold. Taxonomic annotation of representative sequences was accomplished using the SILVA 138 bacterial database [28], providing a detailed classification for each OTU. The sequences obtained from this research were deposited in the Science Data Bank (https://doi.org/10.57760/sciencedb.08303, https://doi.org/10.57760/sciencedb.08311, https://doi.org/10.57760/sciencedb.08312, accessed on 6 January 2023).

A Venn diagram made using the R platform was used to count the number of common and unique ASVs in the multiple samples. Based on the annotation of these OTUs and ASVs, we generated relative abundance profiles at different taxonomic levels. Additionally, we utilised QIIME II software (version 1.9.1) to calculate several alpha diversity indices—including the number of observed features and the Chao1, Shannon, Simpson, Pielou e, and Goods coverage indices [29]—and to plot the rarefaction curves. The hierarchical clustering analysis was evaluated using the unweighted pair group method with the arithmetic mean (UPGMA). The UniFrac distances were calculated and UPGMA sample clustering trees were constructed using QIIME II software (Version 1.9.1) [30]. The results of the PCA were plotted using R software (Version 2.15.3) [31], and the linear discriminant analysis (LDA) effect size (LEfSe) method (http://huttenhower.sph.harvard.edu/lefse/ (accessed on 12 September 2024)) was carried out using LEfSe software (LDA Score ≥ 2) [32]. We used redundancy analysis (RDA) to quantify and examine the effects of the total phosphorus (TP), available phosphorus (AP), and pH in the soil on the relative abundance and diversity of beneficial versus pathogenic bacteria in potato inter-root soils [33]. Student’s *t*-test was used to calculate the differences among the samples. Statistical significance was set at *p* < 0.05.

#### 2.2.3. Predicting Soil Bacterial Community Functions

Functional prediction of the bacterial 16S rRNA was performed using FAPROTAX to annotate the ASVs with functional information [34]. Most annotated species could be assigned to groups involved in metabolic or other ecologically relevant functions, such as the biogeochemical cycling of C, N, and S and the degradation of organic compounds [35,36]. The number of ASVs involved in specific functions and their relative abundances were calculated [37].

### 2.3. Estimation of the Relative Abundance of the txtA Gene in the Soil

To assess the number of CS pathogens in rhizosphere soil samples, qRT-PCR was used to quantify the expression of the *txtA* gene (genes for thaxtomin A biosynthesis). This gene elicits CS symptoms and is located on a conserved and mobile pathogenicity island in pathogenic *Streptomyces* spp. [38]. The *txtA* gene can serve as a marker for pathogenic *Streptomyces* spp. [39]. The soil DNA was extracted using a HiPure Soil DNA Kit (Magen, Guangzhou, China). The online Primer-BLAST tool from NCBI was used to design primers based on the *S. scabiei txtA* gene (GenBank: FN554889.1) for amplification on a CFX96 Touch qRT-PCR Detection System (Bio-Rad, Hercules, CA, USA). The sequence of the forward primer was 5′-CACGTACGCGCAGTTCAATG-3′ and the sequence of the reverse primer was 5′-AGATGATGTAGGCGGGAC-3′ [40].

All qRT PCR products were analysed on a CFX96 Touch instrument (Bio-Rad, Hercules, CA, USA) with 3 technical replicates and 3 biological replicates.

### 2.4. Data Analysis Software

The data employed were collated using Microsoft Excel 2019. SPSS (version 25.0) was used for one-way ANOVA and Pearson’s correlation analysis. CANOCO 5.0 software was used for redundancy analysis (RDA). An unpaired two-tailed Student’s *t*-test (two-group comparison) was carried out to determine the statistical significance using GraphPad Prism software (version 9.3.0). We considered *p*-values < 0.05 as significant; * *p* < 0.05; ** *p* < 0.01; *** *p* < 0.001; **** *p* < 0.0001. Finally, Origin 2018 software was used to graph the data. The data are shown as the means ± SDs.

## 3. Results

### 3.1. Impact of Different Phosphorus Fertiliser Application Rates on Soil α-Diversity

The rhizosphere bacterial communities of potatoes treated with different phosphate fertiliser application rates were analysed based on the 16S rRNA amplicon sequencing method. After denoising the clean data using DADA2, the final ASVs were obtained. The flattened dilution curve (Figure S1a–c) indicates that the bacterial sequencing depth could cover all bacterial species in the soil.

**Figure 1:**
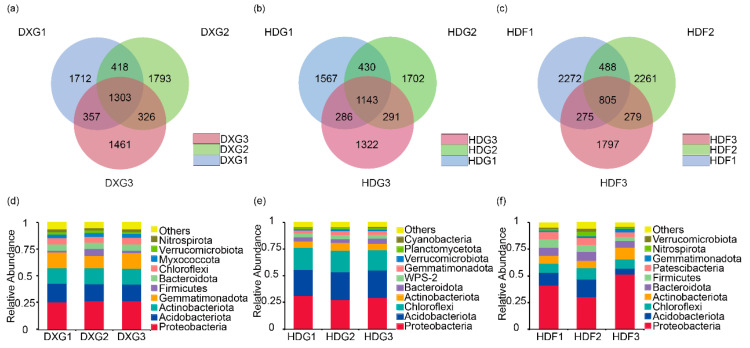
The Venn diagrams (**a**–**c**) illustrate that lower phosphorus fertiliser application rates were beneficial for maintaining bacterial community diversity ((**a**) DXG, (**b**) HDG, and (**c**) HDF). The graphs show the relative abundances of the bacterial community at the phylum level in the potato rhizosphere under different phosphorus fertiliser application rate conditions ((**d**) DXG, (**e**) HDG, and (**f**) HDF).

Based on 16S rRNA amplicon sequencing, the bacterial communities in the potato rhizosphere were analysed under different phosphorus fertiliser application rates. As seen in Figure 1, averages of 7370, 6741, and 8177 bacterial characteristic sequences were obtained in the DXG, HDG, and HDF soil samples, respectively. There were more characteristic sequences in the field, and the microbial diversity was richer. With increases in the phosphorus fertiliser application, the characteristic series initially showed an increasing trend and then gradually decreased and reached a minimum value under high-phosphorus conditions. Notably, the number of characteristic sequences in the soil bacteria of G2 in the potted experiment was significantly higher than those in treatments G1 and G3, whereas, in the field experiment, treatments F1 and F2 resulted in higher numbers of characteristic sequences than treatment F3 did. This indicates that with a lower soil phosphorus fertiliser application rate, the number of soil bacterial content community characteristic sequences increased.

To further elucidate the impact of different phosphorus fertiliser application rates on the diversity and richness of potato rhizosphere soil bacterial communities, we calculated various diversity indices based on the obtained bacterial characteristic sequences. In the DXG and HDG potted experiments, there were no significant differences in the diversity indices under different phosphorus treatments, whereas, in the HDF samples, differences in the observed features and the Chao1, Shannon, Simpson, and Pielou e indices under the phosphorus treatments were significant (*p* < 0.05). It is noteworthy that in the potting experiments, the bacterial community diversity indices were highest under the medium phosphorus application rate. The field trials further validated this trend, with higher Shannon, Simpson, and Pielou e indices observed at moderate phosphorus levels. Conversely, the observed features and Chao1 indices were more abundant under low-phosphorus conditions, suggesting that lower phosphorus fertiliser rates are more beneficial for maintaining soil bacterial community diversity (Table 2). These patterns align with the observed changes in the characteristic sequences, indicating that both excessive and insufficient phosphorus fertiliser applications are detrimental to the preservation of soil bacterial community diversity.

To understand the composition of potato rhizosphere soil bacterial communities under different phosphorus fertiliser application rates, we compared the distribution characteristics of the soil bacterial communities at the phylum level (Figure 1d–f). The predominant bacterial phyla in the three types of soil samples under various phosphorus fertiliser treatments included Proteobacteria, Acidobacteriota, Chloroflexi, Actinobacteriota, Bacteroidota, Gemmatimonadota, and Verrucomicrobiota, which collectively represented > 75% of all sequences. Proteobacteria, Acidobacteriota, Chloroflexi, and Actinobacteriota were the most abundant phyla in almost all of the soil samples. The relative abundance of Acidobacteriota decreased with the increase in the phosphorus application rate in the field experiment with HDF (Figure 1f) but remained almost unchanged in the pot experiment with DXG (Figure 1d) and HDG (Figure 1e). In addition, the relative abundance of Proteobacteria and Verrucomicrobiota varied greatly among treatments. This indicates that the change trend of the dominant bacterial phylum is different under different phosphate fertiliser treatments.

### 3.2. Effects of Phosphorus Fertiliser Application on Soil Bacterial Community Structure

**Figure 2:**
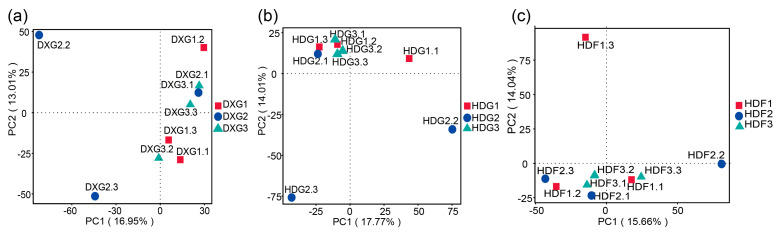
PCA analysis of potato rhizosphere soil bacterial communities under different phosphate fertiliser rates ((**a**) DXG, (**b**) HDG, and (**c**) HDF).

Principal component analysis (PCA) revealed changes in the bacterial community structure among the samples, with significant differences among the groups. Some community differentiation was presented among the treatments under different phosphate fertiliser application rates (Figure 2a–c). In the DXG samples (Figure 2a) under different phosphorus fertiliser treatments, the species differences among the DXG2, DXG1, and DXG3 samples were significant. In the HDG samples (Figure 2b), the trend of species similarity of the samples was consistent with that of the DXG soil samples, suggesting that the bacterial community composition was structurally more similar between the G1 and G3 treatments in the pot experiment. In the HDF samples (Figure 2c), the species differences among the HDF1, HDF2, and HDF3 samples were larger, suggesting that the bacterial communities under the F2 treatment were more similar in compositional structure to those under the F3 treatment in the field trial. Moreover, the comparison of the soil bacterial community structures, as explained through PC1 and PC2, showed that phosphorus fertiliser had a greater impact in the pot experiments than in the field trials.

### 3.3. Correlation Analysis of TP, AP, and pH with Soil Bacterial Community Under Different Phosphorus Fertiliser Application Rates

**Figure 3:**
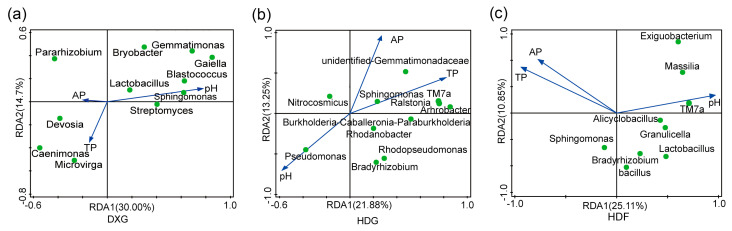
Correlation between potato rhizosphere bacterial community and environmental factors with different phosphorus fertiliser application rates ((**a**) DXG, (**b**) HDG, and (**c**) HDF). Green dots represent bacterial communities.

To analyse the correlation between different phosphorus fertiliser application rates and potato rhizosphere soil bacterial communities, RDA was conducted to examine the relationship between the relative abundance of microbial communities in the three soil samples and the AP, TP, and pH. Among them, in the DXG treatment (Figure 3a), *Bryobacter*, *Gemmatimonas*, and *Blastococcus* showed negative correlations with the TP and AP values and a positive correlation with the pH values. Similarly, in the HDG treatment, *Rhodopseudomonas*, *Bradyrhizobium*, and *Rhodanobacter* exhibited negative correlations with the TP and AP and a positive correlation with the pH. In the HDF treatment (Figure 3c), *Lactobacillus*, *Bradyrhizobium*, *Massilia*, *Sphingomonas*, and *Bacillus* also demonstrated negative correlations with the TP and AP and a positive correlation with pH. It is noteworthy that in the HDG treatment (Figure 3b), *Ralstonia* and *TM7a* showed positive correlations with the TP. In summary, total phosphorus (TP), available phosphorus (AP), and pH are significant environmental factors that influence the composition of bacterial communities.

### 3.4. Prediction of Bacterial Community Function in Potato Rhizosphere Soil Under Different Phosphorus Fertiliser Rates

**Figure 4:**
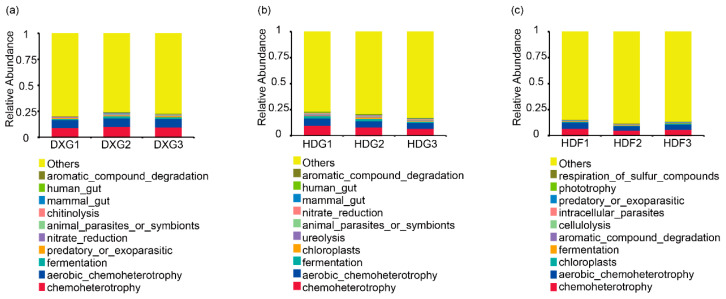
Prediction of bacterial community function in potato rhizosphere soil under different phosphorus fertiliser application rates ((**a**) DXG, (**b**) HDG, and (**c**) HDF).

Changes in the soil bacterial community may lead to changes in its overall metabolic function. Functional annotation of the prokaryotic taxa using FAPROTAX predicted the biogeochemical cycling processes of bacterial communities in potato rhizosphere soil under different phosphorus fertiliser rates. As shown in Figure 4, the results indicate that the top 10 relatively abundant functional groups detected in the three soil samples included chemoheterotrophy, nitrification, hydrocarbon degradation, etc. In the DXG soil sample (Figure 4a), under different phosphorus levels, chemoheterotrophy, aerobic chemoheterotrophy, and fermentation had the highest relative abundances in the moderate phosphorus treatment. In the HDG soil sample (Figure 4b), chemoheterotrophy, aerobic chemoheterotrophy, and fermentation had the highest relative abundances under the low-phosphorus treatment, whereas the relative abundance of aerobic ammonia oxidation tended to increase with increasing phosphorus application rates. In the HDF soil sample (Figure 4c), chemoheterotrophy and aerobic chemoheterotrophy had the highest relative abundances under the moderate phosphorus treatment. The relative abundance of chemoheterotrophy, aerobic chemoheterotrophy, fermentation, ureolysis, and animal parasites or symbionts decreased with the increase in phosphate fertiliser. Overall, lower phosphorus fertiliser application rates were conducive to maintaining the relative abundance of the main functional groups in the cycling mode, with chemoheterotrophy being the main metabolic pattern of the bacterial community in the potato rhizosphere soil, followed by aerobic chemoheterotrophy, fermentation, aromatic compound degradation, and nitrate reduction. These patterns were present in the soils from both Dingxi and Huidong, indicating that bacterial communities actively participate in the biogeochemical cycling processes of carbon, hydrogen, nitrogen, and other elements in potato rhizosphere soil.

### 3.5. Effects of Phosphorus Fertiliser Application on Potato Scab Severity, Relative Abundance of Pathogenic Streptomyces sp., and Growth Indices

**Figure 5:**
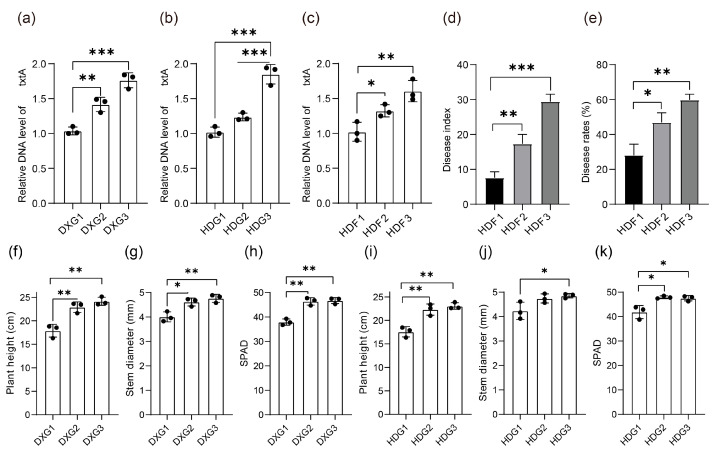
The relative abundance of *txtA* in pathogenic *Streptomyces* in potatoes under different phosphorus application rates and the incidence of potato CS in HDF. The relative abundances of *txtA* in (**a**) DXG, (**b**) HDG, and (**c**) HDF; (**d**) the incidence of CS disease in HDF; (**e**) disease severity index in HDF; (**f**) plant height in DXG; (**g**) stem diameter in DXG; (**h**) SPAD in DXG; (**i**) plant height in HDG; (**j**) stem diameter in HDG; (**k**) SPAD in HDG. * Significant at *p* < 0.05, ** significant at *p <* 0.01, and *** significant at *p <* 0.001.

To investigate the occurrence of potato CS under different phosphorus fertiliser levels, *txtA* is often used to detect and analyse the relative abundance of pathogenic *Streptomyces* in the rhizosphere of potatoes. Therefore, the relative abundance of *txtA* in the soil samples from each treatment was statistically analysed. As shown in Figure 5a–c, the differences in the biomass of *txtA* among the different soil samples were significant (*p* < 0.05). The differences reached extremely significant levels in the different soil samples in the pot experiment under the high-phosphorus treatment (*p* < 0.01). Furthermore, we carried out detailed measurements of the plant height, stem diameter, and relative chlorophyll content of potatoes at the tuberisation stage in the pot experiment (Figure 5f–k). The results show that the potato plant height and stem diameter were significantly lower (*p* < 0.05) than those of the medium- and high-phosphorus-treated controls in both the DXG and HDG potting trials. In addition, we observed that the leaves of the potato plants under the low-phosphorus treatment exhibited yellowing (Figure S4), which was consistent with the significant decreasing trend of the relative chlorophyll content. In contrast, the potato plants under the medium- and high-phosphorus treatments showed similar performance in terms of plant height, stem diameter, and relative chlorophyll content, which were not significantly different (*p* > 0.05). This suggests that increasing the phosphorus fertiliser application rate within a certain range can significantly promote potato growth and chlorophyll synthesis, but beyond a certain threshold, further increases in the phosphorus fertiliser application rate have a limited effect on potato growth promotion.

In a field trial field in Huidong, we randomly selected 60 potatoes for statistical investigation of potato scab disease (Table S2). Accordingly, the CS incidence rates of HDF1, HDF2 and HDF3 were calculated to be 28.33%, 46.66%, and 59.44%, respectively, corresponding to disease severity indices of 7.66, 17.33, and 29.44, respectively (Figure 5e). The differences among the treatments reached significant and highly significant levels, which were consistent with the results of the relative contents of the *txtA* gene, further confirming that phosphorus fertiliser had a significant effect on the relative abundance of pathogenic *Streptomyces* spp. in potato (*p* < 0.05). In addition, measurements were carried out on the incidence of potato scab in the field trial (Figure S5), which was found to have gradually increased with the increases in the phosphorus fertiliser application rate.

### 3.6. Impact of Phosphorus Fertiliser Application Rate on the Relative Abundance of Antagonistic Bacteria Against Pathogenic Streptomyces

**Figure 6:**
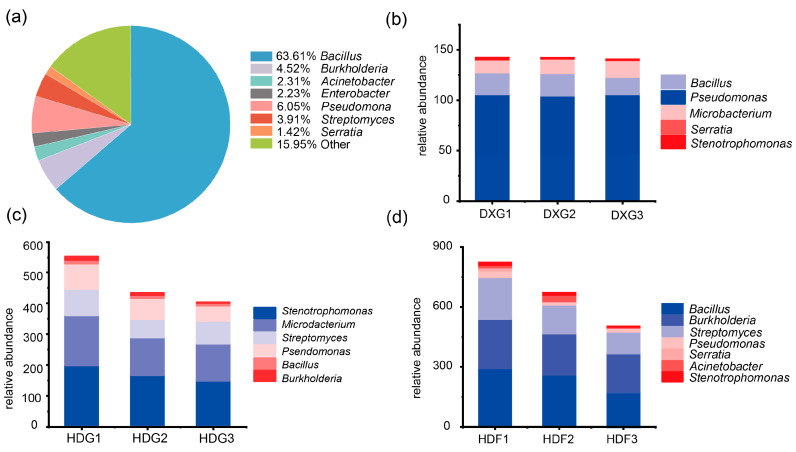
Phosphate fertiliser application rate effects on the abundance of antagonistic bacteria against *S. scabies*. (**a**) A pie chart depicting the distribution of major antagonistic bacterial genera in the potato rhizosphere. Stacked bar charts at the genus level showing the relative abundance of antagonistic bacteria ((**b**) DXG, (**c**) HDG, (**d**) HDF).

We identified 1124 bacteria with antagonistic bacteria against *S. scabies* from field soils of potatoes grown in different regions of China (Figure 6a). The predominant antagonistic bacteria were *Bacillus* (55.0%), *Acinetobacter* (22%), *Pseudomonas* (8.62%), *Burkholderia* (4.52%), *Streptomyces* (3.49%), and *Serratia* (0.3%). Analysis based on high-throughput sequencing of the 16S rRNA gene amplicons revealed that with increases in the phosphorus fertiliser application rate, the relative abundance of major antagonistic bacterial genera—such as *Bacillus*, *Pseudomonas*, *Serratia*, and *Burkholderia*—decreased to varying degrees in the HDF (Figure 6b) and HDG (Figure 6c). In the HDF sample (Figure 6d), the relative abundance of *Acinetobacter* showed a significant decreasing trend. In the DXG, the abundances of *Pseudomonas* and *Serratia* significantly decreased, whereas the relative abundances of other antagonistic bacterial genera did not show significant changes.

**Figure 7:**
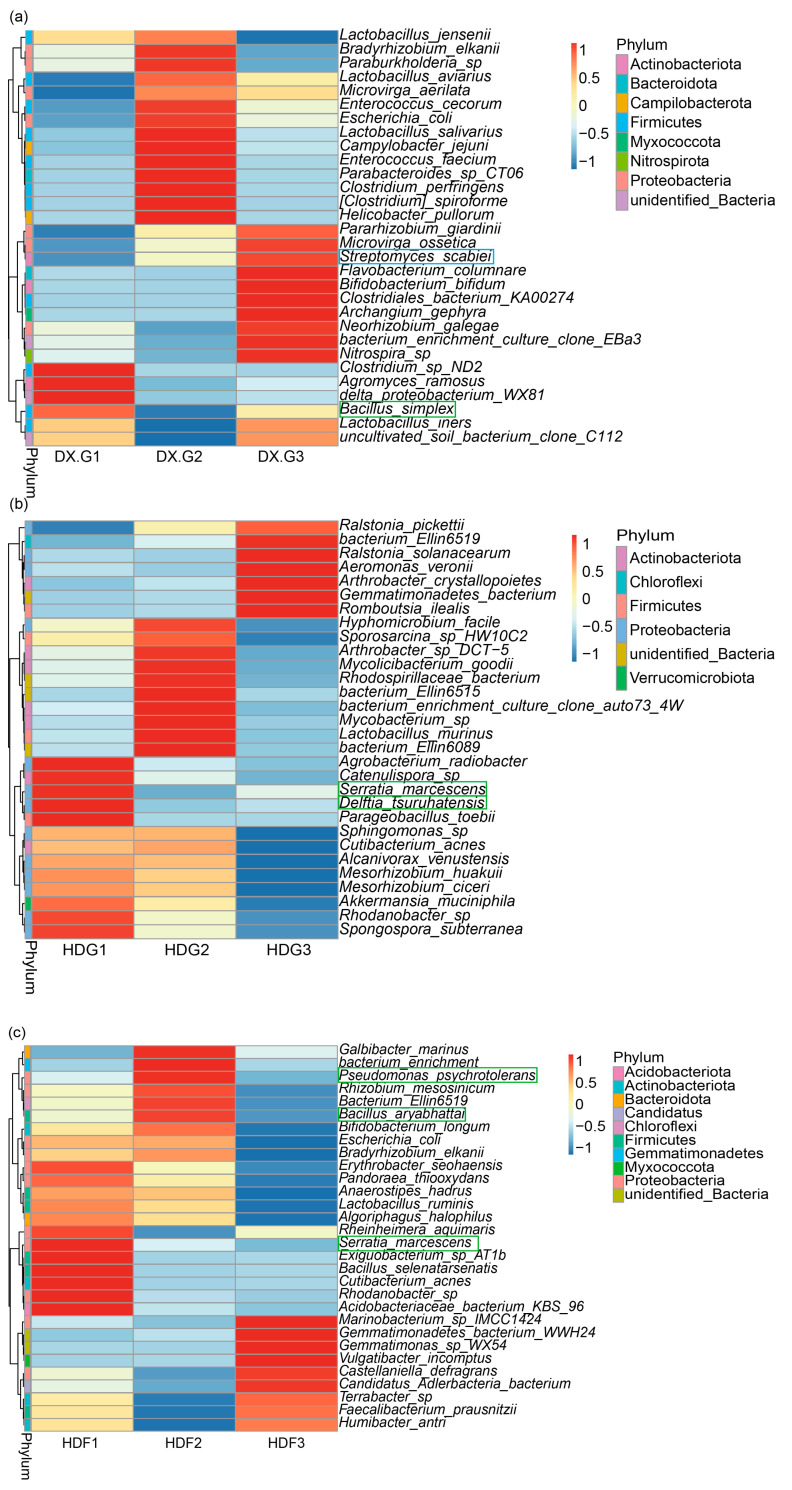
Clustered heat map of relative abundances of the top 30 bacterial species at the species level ((**a**) DXG, (**b**) HDG, and (**c**) HDF). The green boxes mark the antagonistic bacteria and the blue boxes mark the pathogenic bacteria. The chromatograms, from red to blue, indicate a gradual decrease in the abundance of bacterial populations.

Comparing the antagonistic bacteria, we screened and further analysed the top 30 bacterial genera in relative abundance in the soil samples. We found that *Bacillus simplex* in DXG (Figure 7a), *Serratia marcescens* and *Delftia tsuruhatensis* in HDG (Figure 7b), and *Pseudomonas psychrotoleroms*, *Bacillus aryabhatttai*, and *Serratia marcescens* in HDF (Figure 7c) showed significant downward trends. At the same time, the antagonistic effect was verified (Figure S2). In particular, an increase in the relative abundance of pathogenic potato *streptomycetes* spp. was detected in DXG with the increasing phosphate fertiliser application rates (Figure 7a), which is consistent with the results of the LEfSe analysis (Figure S3).

## 4. Discussion

### 4.1. Lower Phosphorus Fertiliser Application Rates Were More Conducive to Maintaining Soil Bacterial Community Diversity and the Relative Abundance of Key Functional Groups in the Major Cycling Patterns

Increased phosphorus fertiliser application altered the composition of the potato soil bacterial community [41] (Figure 1d–f), leading to a decrease in the number of bacterial ASVs (Figure 1a–c). Excessive application of phosphorus fertiliser is detrimental to maintaining soil bacterial community diversity; for example, high-phosphorus fertiliser treatments significantly reduced the diversity of the inter-root bacterial community in wheat [42]. In the HDF field trial, the differences in the observed features and Chao1, Shannon, Simpson, and Pielou e indices among the treatments with different phosphorus fertiliser application rates reached significant levels; however, in the DXG and HDG pot trials, there were no significant differences in the α diversity indices among the samples, probably due to the different external environments for plant growth [43]. In contrast, an increased application rate of phosphorus fertiliser in a pasture significantly increased the bacterial diversity [44], which was contrary to our findings; this was probably because the grassland had not been fertilised for a long time, which led to differences in the basal phosphorus fertiliser content of the soil samples (Table S1) that in turn affected the diversity of the soil bacterial community. Lower phosphorus fertiliser application rates are advantageous for maintaining bacterial diversity because they supply essential nutrients without causing nutrient overload [45], which mitigates competition among microorganisms. This balanced nutrient provision fosters ecological niche differentiation, thereby enhancing bacterial resilience and adaptability [46]. Consequently, it contributes to a more stable and diverse microbial community.

The diversity and composition of microbial communities are directly associated with their functions [47], and the use of phosphorus fertiliser can significantly affect the structure and function of microbial communities in the soil. As indicated by the results of the FAPROTAX analysis, chemoheterotrophy is the main metabolic mode of the potato rhizosphere soil bacterial community. It does not rely on light energy [48] but is a cyclic mode that uses organic matter as an energy and carbon source, which suggests that a large number of bacteria in the soil obtain carbon and energy from decomposing organic matter [49]. Among these cycling patterns, nitrate reduction had higher relative abundance under the lower phosphorus fertiliser treatments, suggesting that the bacterial community in the soil samples had a high metabolic potential for N cycling [50]. The major functional groups of temperate forest soils were chemoheterotrophy and aerobic chemoheterotrophy [51], which is also consistent with the results of our soil bacterial community function prediction study, providing further evidence that these functions are broad ecosystem functions [52] covering a wide range of activities such as decomposing organic matter, nitrogen cycling, and carbon cycling, which are essential features for most microbial growth and metabolic processes.

### 4.2. Different Phosphorus Fertiliser Treatments Altered the Composition and Structure of Bacterial Communities

The analysis of dominant soil bacterial communities at the phylum level revealed that the relative abundance of Actinobacteriota increased with the phosphorus fertiliser rate in HDF and that Actinobacteriota in maize rhizosphere soils was also positively correlated with phosphorus fertiliser application rate [53], possibly since Actinobacteriota mostly has the function of phosphorus solubilisation and its abundance is closely related to quick-acting phosphorus content in soil [54]. The relative abundance of Gemmatimonadota in HDF and DXG was higher under a high-phosphorus treatment, which may be related to the strong adaptability of this kind of bacteria [55]. In all soil samples, the relative abundance of Verrucomicrobiota was the lowest under a high-phosphorus treatment. However, the relative abundance of Verrucomicrobiota increased in black soil under long-term phosphorus fertiliser treatments [56], which may be related to differences in the soil texture and pH.

PCA analysis (Figure 2) showed that the effects of phosphorus fertiliser on bacterial community structure were more significant under the potting conditions than in the field experiment. This may be due to the complexity of the field environment, in which multiple factors, such as soil pH [57], come together, of which phosphorus fertiliser application is only one. In contrast, the potting environment was more controlled, with higher soil type consistency and more closed nutrient cycling [58], thus making the microbial response to the phosphorus fertiliser more sensitive. In addition, the competitive and symbiotic relationships of bacterial communities under pot conditions may be rapidly adjusted through phosphorus fertiliser application [59], while diverse factors in the field environment may mask this effect.

The results of the redundancy analysis (RDA) revealed that in the DXG treatment (Figure 3a), the relative abundance of *Bryobacter* spp. decreased with the increasing phosphorus fertiliser application rates. These bacteria typically play a key role in soil nitrogen cycling, enhancing soil phosphorus use efficiency and the decomposition of organic matter [60]. In the HDF treatment (Figure 3c), the relative abundance of the genus *Massilia* increased under low-phosphorus conditions, possibly because they can effectively solubilise phosphates in nutrient-limited environments, thereby enhancing the availability of phosphorus in the soil [61]. Notably, *Bacillus* spp. were found to promote plant growth and development and suppress plant pathogens [62,63,64,65], yet our study found a negative correlation between the relative abundance and the phosphorus fertiliser application rate (Figure 3c). In the HDG treatment (Figure 3b), the addition of phosphate fertiliser increased the relative abundance of *Ralstonia* spp., of which there are pathogenic strains such as *Ralstonia* solanacearum, which can colonise the vascular system of plant roots. This colonisation can lead to various plant infections and ultimately cause plant wilting and even death [66,67]. This phenomenon suggests that a high phosphorus fertiliser application rate may increase the number of harmful bacteria in the rhizosphere soil of potatoes while decreasing the microbial diversity and the relative abundance of bacteria beneficial to plant health.

### 4.3. The Phosphorus Fertiliser Application Rate Was Positively Correlated with the Relative Abundance of the txtA Gene in the Rhizospheric Soil, Whereas It Was Negatively Correlated with the Relative Abundance of Antagonistic Bacteria of Pathogenic Streptomyces

*S. scabiei* was detected in the DXG3 soil samples, a finding that points to a significant increase in the abundance of pathogenic *Streptomyces* under high-phosphorus treatment conditions (Figure S3a). This phenomenon aligns with prior research indicating that elevated phosphorus levels in the soil can foster the proliferation and activity of pathogenic microorganisms, thereby increasing the susceptibility of plants to diseases. The *txtA* gene—a key biomarker of *Streptomyces* pathogenicity—was further investigated, revealing a significant positive correlation between changes in its biomass in the soil and the amount of phosphorus fertiliser applied (Figure 5a–c). This is consistent with the statistical results of potato tuber disease severity in the field trials (Figure 5e,f), i.e., phosphorus accumulation aggravates the occurrence of potato CS [18]. Consequently, the number of pathogenic bacteria increased and the number of antagonistic bacteria decreased in soil samples treated with high phosphorus, which is not conducive to the maintenance of a microecological environment for healthy growth in potato fields. In particular, the proliferation of pathogenic *Streptomyces* may have exacerbated its ability to infest the host plant under high-phosphorus conditions, which is consistent with the effects of soil nutrient changes on the microbial community structure and function. There are a few reports on the effect of phosphorus fertiliser on soil-borne diseases, including CS of potato, and most studies have focused more on the correlation between the phosphorus fertiliser application rate and potato growth and yield. Among them, there was a significant positive correlation between the phosphorus fertiliser application rate and potato yield [68], and the highest potato yield was obtained at a phosphorus fertiliser rate of 150 kg P_2_O_5_ ha^−1^ [69]; however, the long-term application of phosphorus fertiliser in large quantities, although it may increase yield, ignores the possibility of exacerbating soil diseases.

Referring to our screening of antagonistic strains of potato pathogenic *Streptomyces scabies*, it was found that the antagonistic strains of *Streptomyces scabies* in the soil samples from different habitats showed decreasing trends with the increasing fertiliser application rates (Figure 6b,d). The presence of *Streptomyces scabies* antagonists is shown in clustered heat maps of the relative abundance of the top 30 bacterial species at the species level (Figure 7a–c). *Bacillus simplex* promotes the growth and development of a wide range of crops [70,71], and *Serratia marcescens* in the HDG has been shown to perform biological control, reducing the use of chemical pesticides and environmental pollution as well as helping to promote soil health and ecological balance [72]. *Delftia tsuruhatensis* is present in the rhizosphere environment of plants and can increase nutrient availability in the soil and promote plant growth and development [73,74]. Bacillus aryabhatttai is reduced in HDF, but it degrades pollutants and helps to protect the health of the ecosystem [74,75]. In conclusion, high phosphorus fertiliser dosage affects the population and function of beneficial microorganisms and exacerbates the problem of soil contamination, thus adversely affecting soil health.

Therefore, improving the economic benefits of potatoes and promoting sustainable green development to analyse the impact of the phosphate fertiliser application rate on the bacterial community in potato rhizosphere soil and the abundance of major soil-borne pathogens is of great significance. In particular, the use of antagonistic bacteria with phosphorus-solubilising capabilities to suppress the damage caused by potato CS is a topic worthy of the attention of future researchers.

## 5. Conclusions

The differences in the dominant bacterial communities in diseased potato rhizosphere soil indicate that the phosphorus fertiliser application rate has a significant influence on the bacterial community’s distribution and the severity of potato CS in the field. The trends in the changes in the dominant phyla, Actinobacteriota, Verrucomicrobiota, and Chloroflexi, varied in different soil samples with different phosphorus fertiliser application rates, possibly because of variations in the external environmental factors in different habitats. Additionally, the relative abundance of pathogenic *Streptomyces* spp. was positively correlated with the phosphorus fertiliser application rate, whereas the relative abundance of antagonistic bacteria showed a significant downward trend with an increasing phosphorus fertiliser application rate. The functional prediction results also indicate that lower phosphorus fertiliser application rates are more conducive to maintaining the relative abundances of major functional groups. Therefore, increasing the phosphorus fertiliser application rate affected the diversity of bacterial communities in the potato rhizosphere soil, altered their community structure and functional roles in biochemical cycling processes, increased the severity of potato CS, and decreased the relative abundance of antagonistic bacteria against *S. scabies*.

## Figures and Tables

**Table 1:** Amount of fertiliser applied for each treatment (kg/ha).

Treatment (Fertiliser Applied)	G1 (F1)	G2 (F2)	G3 (F3)
P_2_O_5_	0	120	240
N	240	240	240
K_2_O	240	240	240

Note: G1 (F1) denotes the low-phosphorus treatment; G2 (F2) denotes the medium phosphorus treatment; G3 (F3) denotes the high-phosphorus treatment.

**Table 2:** Alpha diversity of bacterial communities in potato rhizosphere soil across varying phosphorus fertiliser rates.

Treatment	Observed Features	Chao1	Shannon	Simpson	Pielou e	Goods Coverage
DXG1	3790 ± 110.39	3813.33 ± 120.07	10.23 ± 0.10	0.998 ± 0.001	0.860 ± 0.01	0.999 ± 0.001
DXG2	3840 ± 167.51	3879.77 ± 171.11	10.42 ± 0.14 *	0.998 ± 0.001	0.875 ± 0.01	0.999 ± 0.001
DXG3	3447 ± 104.71	3482.88 ± 108.94	10.06 ± 0.04 *	0.998 ± 0.001	0.856 ± 0.01	0.999 ± 0.001
HDG1	3426 ± 127.24	3478.50 ± 129.33	9.54 ± 0.15	0.996 ± 0.001	0.812 ± 0.01	0.999 ± 0.001
HDG2	3566 ± 110.19	3604.28 ± 112.28	9.73 ± 0.21	0.997 ± 0.001	0.824 ± 0.01	0.999 ± 0.00
HDG3	3042 ± 134.08	3058.31 ± 135.40	9.36 ± 0.48	0.995 ± 0.001	0.809 ± 0.01	0.999 ± 0.001
HDF1	3840 ± 138.35	3925.92 ± 142.02	9.76 ± 0.27	0.994 ± 0.002	0.819 ± 0.02	0.997 ± 1.001
HDF2	3833 ± 54.88 *	3923.62 ± 56.05 *	10.13 ± 0.97 **	0.997 ± 0.002 **	0.851 ± 0.01 **	0.997 ± 0.001
HDF3	3156 ± 28.02 *	3246.62 ± 48.37 *	8.75 ± 0.21 *	0.982 ± 0.003 *	0.753 ± 0.02 *	0.997 ± 0.001

* Data shown are means and standard deviations. Statistically significant differences among treatments are marked with asterisk(s): ** *p* < 0.01; * *p* < 0.05.

## Data Availability

The raw read data were submitted to the Science Data Bank (https://doi.org/10.57760/sciencedb.08303, https://doi.org/10.57760/sciencedb.08311, https://doi.org/10.57760/sciencedb.08312, accessed on 6 January 2023).

## Supplementary Materials

The following supporting information can be downloaded at https://www.mdpi.com/article/10.3390/microorganisms12112322/s1, Figure S1: Dilution profiles of inter-root soil bacterial communities of potato at different phosphate fertiliser rates ((a) DXG, (b) HDG, and (c) HDF); Figure S2: The inhibitory effects of the target strain on pathogenic *Streptomyces* ((a) only coated with *S. scabies*, (b) inhibition zone of *Bacillus simplex*, (c) inhibition zone of *Serratia marcescens*, (d) inhibition zone of *Delftia tsuruhatensis*, (e) inhibition zone of *Pseudomonas psychrotoleroms*, and (f) inhibition zone of *Bacillus aryabhatttai*); Figure S3: The differential groups of bacteria at different taxonomic levels (LDA ≥ 2.0) ((a) DXG, (b) HDG, and (c) HDF). The evolutionary branching diagram of LEfSe analysis shows the different compositions of bacterial taxa ((d) DXG, (e) HDG, and (f) HDF). **Figure S4**: The effects of different rates of phosphorus fertiliser on the phenotype of pot potatoes at the tillering stage. Figure S5: The effects of different rates of phosphate fertiliser on the extent of potato scab in the field trials. Table S1: Physico-chemical properties of the reference soil samples before planting. Table S2: Statistics on potato scab in field trials.
