# Supplementary material for: Deciphering the Effects of Phosphate Fertiliser on Rhizospheric Bacterial Community Structure and Potato Common Scab
Source: Microorganisms. 2024 Nov 15;12(11):2322. doi: 10.3390/microorganisms12112322 (PMC11596807; doi:10.3390/microorganisms12112322)
Supplement: Supplementary file 1 [file microorganisms-12-02322-s001.zip › microorganisms-3303939-supplementary.pdf]

Deciphering the effects of phosphate fertiliser on rhizospheric bacterial community structure and potato common scab

The following supporting information is provided for this article, including 5 figures and 2 table.

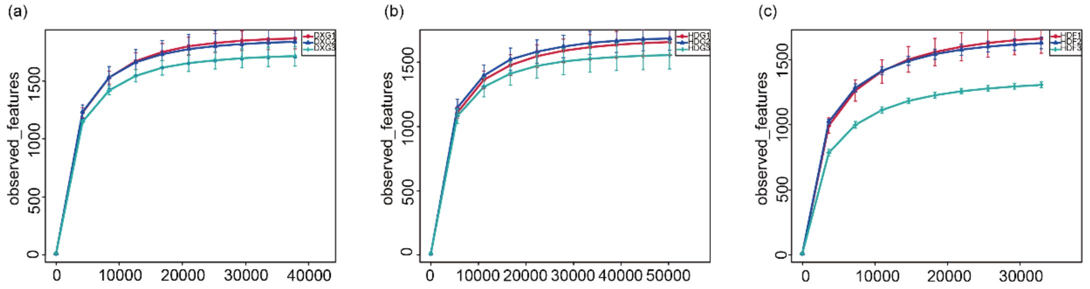

Figure.S1: Dilution profiles of inter-root soil bacterial communities of potato at different phosphate fertiliser rates (a for DXG, b for HDG, c for HDF)

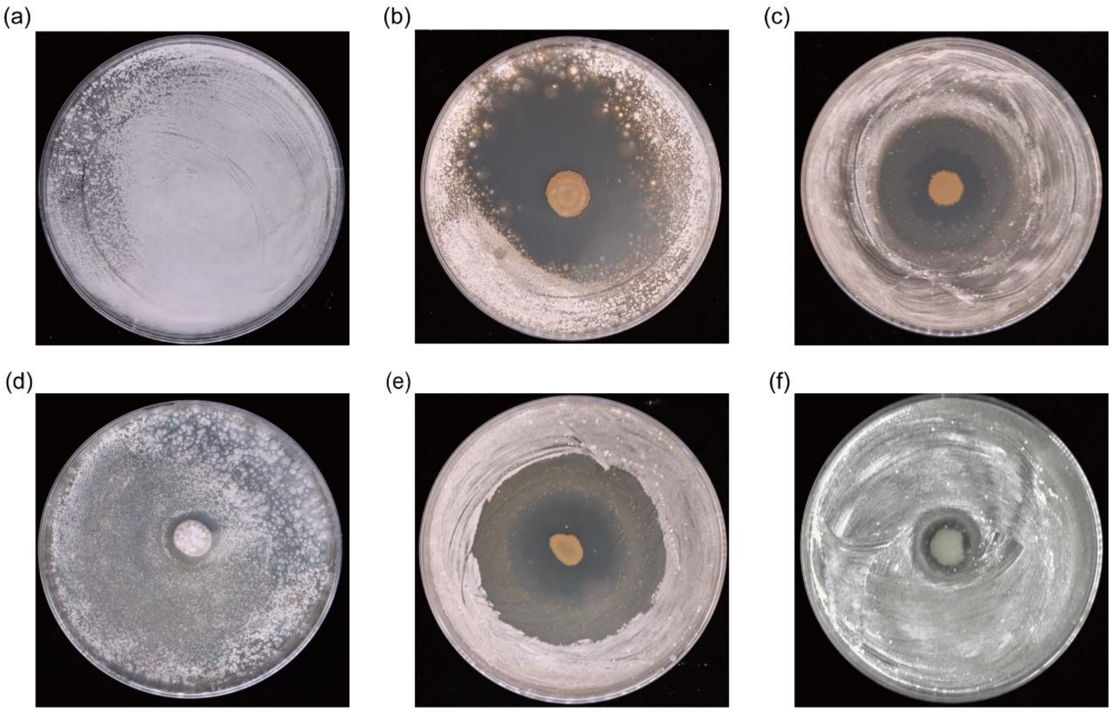

Figure.S2: The inhibitory effect of the target strain on pathogenic Streptomyces (a only coated with *S. scabies*, b inhibition zone of *Bacillus simplex*, c inhibition zone of *Serratia marcescens*, d inhibition zone of *Delftia tsuruhatensis*, e inhibition zone of *Pseudomonas psychrotoleroms*, f inhibition zone of *Bacillus aryabhatttai*.)

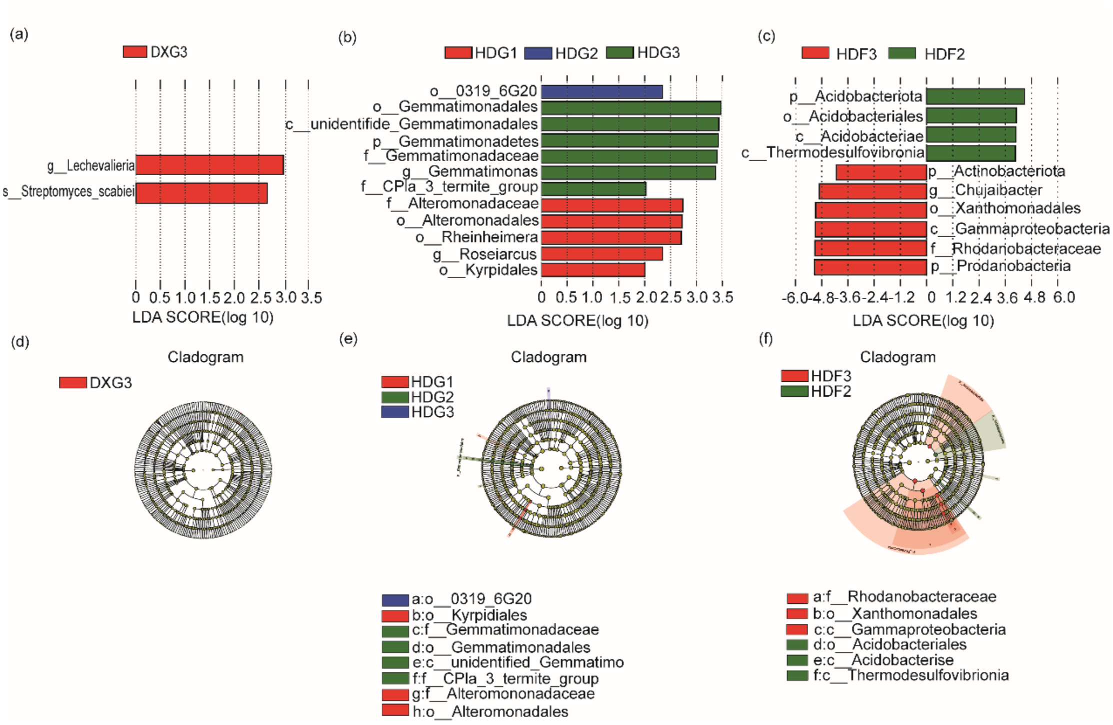

Figure.S3: The differential groups of bacteria at different taxonomic levels (LDA≥2.0) (a for DXG, b for HDG, c for HDF) The evolutionary branching diagram of LEfSe analysis shows the composi-tion of bacterial differential taxa (d for DXG, e for HDG, f for HDF).

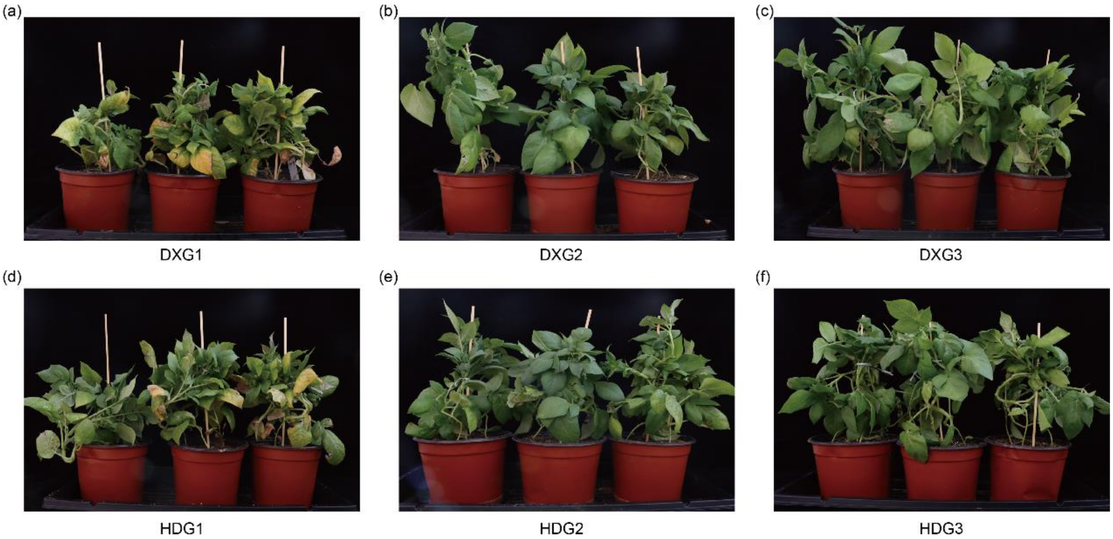

Figure.S4: The effects of different rates of phosphorus fertiliser on the phenotype of pot potatoes

at the tuberisation stage

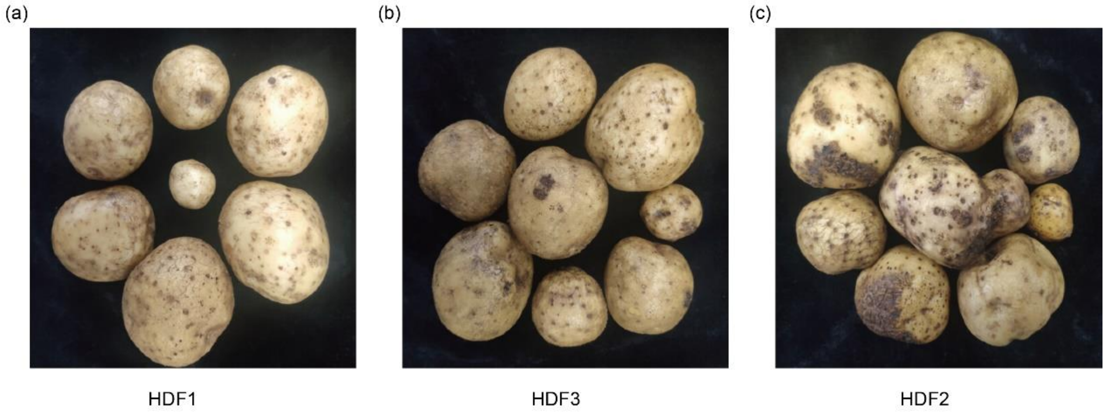

Figure.S5: The effects of different rates of phosphate fertiliser on the extent of potato scab in the field trials.

Table S1: Physico-chemical properties of the reference soil samples before planting

Location	pH	TP(g/kg)	AP(g/kg)	TN(g/kg)	AN(g/kg)	TK(g/kg)	AK(g/kg)	SOM(g/kg)
DX soil	8.10	0.668	0.029	0.822	0.0552	15.1	0.0983	15.10
HD soil	4.67	1.31	0.101	2.01	0.696	18.15	0.449	32.4

Table S2: Statistics on potato scab in field trials

treatment	number of grains	sickness						Disease rate(%)	Disease index	Average incidence rate	Average disease index
		0	1	2	3	4	5				
HDF1.1	60	47	10	2	1	0	0	21.66	5.67		
HDF1.2	60	43	12	3	2	0	0	28.33	8.00	28.33	7.76
HDF1.3	60	39	16	3	2	0	0	35.00	9.33		
HDF2.1	60	37	10	7	3	3	0	38.33	15.00		
HDF2.2	60	29	18	4	3	4	2	51.66	20.33	46.67	17.33
HDF2.3	60	30	17	7	5	1	0	50.00	16.67		
HDF3.1	60	23	11	9	7	5	5	61.66	31.67		
HDF3.2	60	26	11	7	6	6	4	56.67	29.00	59.44	29.44
HDF3.3	60	24	14	8	6	5	3	60.00	27.67		
